# Editorial: 2021 WHO classification of pediatric brain tumors: a final wedding between morphology and molecular biology?

**DOI:** 10.3389/fnmol.2024.1423298

**Published:** 2024-05-08

**Authors:** Angela Mastronuzzi, Lucia Quaglietta, Elisabetta Schiavello, Andrea Carai

**Affiliations:** ^1^Hematology/Oncology, Cell Therapy, Gene Therapies and Hemopoietic Transplant, Bambino Gesù Children's Hospital, Instituto di Ricerca e Cura a Carattere Scientifico (IRCCS), Rome, Italy; ^2^Pediatric Neuro-Oncology, Santobono-Pausilipon Children's Hospital, Naples, Italy; ^3^Pediatric Oncology Unit, Fondazione Instituto di Ricerca e Cura a Carattere Scientifico (IRCCS) Istituto Nazionale dei Tumori, Milan, Italy; ^4^Neurosciences, Neurosurgery Unit, Bambino Gesù Children's Hospital, Instituto di Ricerca e Cura a Carattere Scientifico (IRCCS), Rome, Italy

**Keywords:** pediatric brain tumors, WHO CNS5, CNS tumors, pediatric cancer, molecular profiling

This Frontiers Research Topic includes a collection of nine original contributions and reviews on different aspects of pediatric tumors of the central nervous system (CNS), specifically related the World Health Organization (WHO) Classification of Tumors of the Central Nervous System (CNS5) (Louis et al., 2021) and their implication in diagnosis, prognosis, stratification, and target therapies on patients (Fuller et al., 2017; Fangusaro and Bandopadhayay, 2021; Guo et al., 2023; Li et al., 2023).

Skitchenko et al. identified four candidate somatic mutations potentially explaining the medulloblastoma (MB) onset in two pediatric patients and providing new biological insights into the mechanisms of tumor development. Molecular diagnostics for two WNT-MB cases without chromosome 6 monosomy or mutations in CTNNB1 and APC are described.

Vallero et al. reviewed the current literature on H3K27-altered diffuse midline glioma (DMG) and addressed questions such as when additional mutations are found, which one should we focus on in order to make the correct clinical decision. H3K27 status has become a fundamental supplement to the histological grading of pediatric gliomas but not sufficient alone to exhaustively define the complex biological behavior of DMG in children and might not represent an indication for a unique treatment strategy across all patients, irrespective of age, additional molecular alterations, and tumor location. Therefore, each DMG case should have its own unique and precise molecular characterization. The ultimate goal is to treat all patients with a personalized therapy tailored to the specific characteristics of their tumor.

In their review Cipri, Del Baldo et al. described the major molecular alterations detected in pediatric low-grade gliomas (pLGGs) and the molecular target therapy, which is feasible/available to date. Having a better understanding of tumor biology and a germline and somatic genomic approach will play a central role in the therapy strategy of pLGG for the development of increasingly tailored therapies. It cannot be underestimated that limitations still exist, regarding the adverse effects of long-term treatment.

De Martino et al. reported on two pediatric patients affected by DMG with extra-neural dissemination, both showing disease progression at bone sites and partial response of intracranial DMG to second-line treatment with craniospinal irradiation and systemic chemotherapy with irinotecan and bevacizumab regimen. Extra-neural metastasis of DMG is a rare event and no standard therapy exists. Due to its rarity, the biological mechanisms behind tumor dissemination outside the CNS of DMG have not been well-described. Although improved care of patients affected by DMG is going to lead in some cases to longer survival, extra-neural metastases in DMG were detected at diagnosis or relatively early after diagnosis.

The review of Caroleo et al. described an exceptional case of an infant carrying a germline and somatic pathogenic variant of PTEN and a germline and somatic pathogenic variant of CHEK2 who developed a MB SHH in addition to intestinal polyposis. PTEN gene variants often present in childhood with macrocephaly, developmental delay, and/or autism spectrum disorder while tumors and intestinal polyps are commonly detected in adults. PHTS is rarely associated with childhood brain tumors with only two reported cases of MB. Although the association is rare, the panel of genes to be tested in the presence of an MB SHH could be extended to PTEN. To date, the role of CHEK2 remains uncertain. The discovery of a PTEN germline mutation should induce the clinician to promptly provide genetic counseling in order to assess and monitor the occurrence of other PHTS clinical features and set up careful surveillance.

Weiser et al. explained that understanding the longitudinal overlap and glioma evolution from childhood to adulthood is an important research gap. Treatment optimization, including implementation of targeted therapies, starts with the adoption of appropriate molecular testing as part of the diagnostic work-up, for biomarker identification. Even though the molecular features vary between pediatric, adult, and—most likely—adolescent and young adult (AYA) gliomas, these tumors also share common tumorigenic pathways, including overexpression of oncogenes, activation of RTKs, epigenetic dysregulations, and increased metabolic pathways, which should be explored for introducing new therapies in age-inclusive clinical trials. To bridge this gap and offer better treatment options, exchange of expertise and close collaboration between pediatric and adult neuro-oncologists—and broader multidisciplinary clinical teams—is indispensable. Ensuring access to appropriate molecular testing to detect key biomarkers, designing age-inclusive clinical trials for gliomas and creating multidisciplinary teams, bridging the pediatric/adult divide, are some of the many actions needed and being implemented in several centers across the world. Additional factors to be considered include the socioeconomic and mental health burden that AYA patients experience.

In their publication Morgacheva et al. explained a case that highlights need for the implementation of molecular methods, especially tumor DNA methylation, in the diagnosis of CNS neoplasms in children. Pediatric CNS tumors demonstrate clinical and biological diversity and variability in the morphological picture, which can lead to misdiagnosis and wrong therapeutic strategies. Diagnostic challenges can be overcome by using novel technological diagnostic approaches such as DNA and RNA sequencing, RNA expression profiling, fluorescence *in situ* hybridization, and DNA methylation. They stated that their case demonstrates the complexity of diagnosing a CNS tumor in a pediatric patient, which was caused by a non-specific clinical and morphologic picture of the tumor itself, which twice led to misdiagnosis and a wrong therapeutic approach. An additional molecular analysis allowed them to find a potential target for precision therapy, which may be useful in the event of disease progression. In diagnostic cases, at least a complete IHC and first level molecular methods [PCR, fluorescence *in situ* hybridization (FISH)] should be used.

Cipri, Fabozzi et al. demonstrated that tropomyosin receptor kinase inhibitors, such as larotrectinib and entrectinib, have showed high efficacy in pediatric patients, also in CNS tumors carrying alterations in NTRK genes. Additional research is necessary to help us to understand better the mechanism of action of these drugs and to identify biomarkers that can help identify patients who will benefit most from therapy.

d'Amati et al. summarized the major changes in the 2021 WHO CNS5, highlighting for each entity the molecular alterations and other information that are relevant for diagnostic, prognostic, or therapeutic purposes. The rationale of this “molecular classification” is also related to the effective and experimental molecular therapies, targeting some cancer-specific genetic events. Reclassification based on molecular investigations has allowed identification of specific entities that appear homogeneous in their response to treatment and clinical outcomes. These implications highlight the necessity to adopt the new classification when considering therapeutic options (clinical trials, targeted therapies) and discussing prognosis.

## Author contributions

AM: Conceptualization, Writing – original draft, Writing – review & editing. LQ: Conceptualization, Writing – review & editing. ES: Conceptualization, Writing – review & editing. AC: Conceptualization, Writing – review & editing.
